# Efficacy and safety of obinutuzumab as rescue therapy for rituximab-resistant and rituximab-recurrent primary membranous nephropathy: a retrospective cohort study

**DOI:** 10.1515/med-2026-1440

**Published:** 2026-06-15

**Authors:** Li Tian, Huaying Pei, Tianhua Dong, Mengsi Zhou, Shaomei Li, Xi Zhao, Xuzhi Liang, Lingling Xing

**Affiliations:** Department of Nephrology, The Second Hospital of Hebei Medical University, Shijiazhuang, China; Department of Orthopacdic Surgery, The Third Hospital of Hebei Medical University, Shijiazhuang, China; Breast and Thyroid Surgery Department, The Second Hospital of Hebei Medical University, Shijiazhuang, China

**Keywords:** obinutuzumab, membranous nephropathy, CD20 monoclonal antibody, remission, resistant, recurrent

## Abstract

**Objectives:**

With the recognition of membranous nephropathy as an autoimmune disease, targeting B-cells has emerged as an effective therapeutic agent for primary membranous nephropathy (PMN). About up to 40 % of patients fail to respond to rituximab (RTX). Obinutuzumab is a humanized and glycoengineered type II anti-CD20 monoclonal antibody which demonstrates more powerful and prolonged B-cell depletion to rituximab in certain hematologic malignancies. Our aim was to observe the efficacy and safety of Obinutuzumab in rituximab-resistant and recurrent membranous nephropathy and to find the factors associated with a poor response to Obinutuzumab.

**Methods:**

We conducted a retrospective analysis of consecutive patients with biopsy-proven primary membranous nephropathy who received Obinutuzumab as rescue therapy for rituximab-resistant or rituximab-recurrent disease between September 2022 and March 2025. Obinutuzumab was administered as two separate 1 g intravenous infusions over two to four weeks, with a subsequent 1 g infusion at approximately six months in most patients. The treatment regimen was determined by the medical team based on the patient’s clinical condition and laboratory findings. We assessed the following outcomes: clinical remission rate, immunological remission rate, relapse rate, and incidence of adverse events. Additionally, we explored potential factors associated with treatment response.

**Results:**

The mean age at initiation of Obinutuzumab treatment was 46.5 ± 12.0 years. Patients with CKD stages 1–4 were48 %, 24 %, 20 % and 8 % respectively. Renal pathological findings revealed 15 cases of stage I membranous nephropathy, 6 cases of stage II membranous nephropathy, and 4 cases of atypical membranous nephropathy. Of the 25 patients, 20 were identified as rituximab-resistant, while 5 had recurrent disease after an initial response. During a median follow-up of nine months (range 6–30), complete or partial remission of proteinuria was achieved in 20 of the 25 participants (80 %). The time to achieve clinical remission was four months (range 1–14). Among the 21 patients with anti-phospholipase A2 receptor antibody-associated primary membranous nephropathy, 13 simultaneously achieved immunologic remission. Relapse occurred in two patients following a nine-month period off medication. No clinically severe adverse events were observed during the treatment period. Higher baseline proteinuria (p=0.001) and serum creatinine levels (p=0.03) were associated with a poorer response.

**Conclusions:**

Obinutuzumab may represent a promising treatment strategy for rituximab-resistant and recurrent membranous nephropathy, including in cases with severe renal impairment. A poorer response to obinutuzumab may be associated with higher baseline proteinuria and serum creatinine levels.

## Introduction

Membranous nephropathy (MN) is a kidney disease characterized by thickening of the glomerular basement membrane due to the deposition of immune complexes, which often leads to the nephrotic syndrome and may progress to end-stage renal disease. Membranous nephropathy (MN) is a leading cause of nephrotic syndrome (NS) in nondiabetic adults [1]. Up to 40 % of patients with persistent NS develop kidney failure over a period of 10 years [2]. With the recognition of membranous nephropathy as an autoimmune disease, targeting B-cells has emerged as an effective therapeutic agent for primary membranous nephropathy (PMN) [3], [4], [5], [6]. Rituximab (RTX) has become first-line therapy for PMN at moderate or high risk for progression to renal impairment [7].However some researches showed RTX had a failure rate of more than 40 % to treat PMN and the recurrence rate was up to 27 % after treatment [8].

Obinutuzumab, a humanized and glycoengineered type II anti-CD20 monoclonal antibody, induces more potent and sustained B-cell depletion than rituximab in certain hematologic malignancies and B-lymphoproliferative disorders [9], [10], [11], [12]. Its efficacy and safety have been demonstrated in several studies and emerging evidence suggests it may serve as a treatment option for patients with membranous nephropathy who are refractory to or intolerant of rituximab, and potentially even as first-line therapy in newly diagnosed cases [13], [14], [15], [16], [17], [18]. However, data remain limited regarding its use in rituximab-resistant and recurrent primary membranous nephropathy, and factors associated with treatment response have not been well characterized. Therefore, we hypothesized that Obinutuzumab would demonstrate favorable efficacy and an acceptable safety profile in this population, with clinical outcomes potentially influenced by baseline immunological and clinical characteristics. Accordingly, this study aimed to evaluate the efficacy and safety of Obinutuzumab in patients with rituximab-resistant and recurrent primary membranous nephropathy, and to identify factors associated with a poor response.

## Methods

### Patient population

We conducted a retrospective analysis of consecutive patients with biopsy-proven primary membranous nephropathy who received Obinutuzumab as rescue therapy for rituximab-resistant or rituximab-recurrent disease between September 2022 and March 2025. Inclusion criteria were [1]: primary membranous nephropathy (PMN) with a diagnosis of nephrotic syndrome [2], an eGFR greater than 15 mL/min/1.73 m^2^ [3], stable background therapy, including an Sodium-glucose cotransporter 2 (SGLT2) inhibitor and/or Angiotensin II Receptor Blocker, and [4] a history of either failure to respond to RTX or relapse after RTX therapy. Exclusion criteria was the concomitant use of other immunosuppressive agents. Among the 25 patients, 20 were resistant to rituximab, and 5 relapsed after achieving a partial response, with a median follow-up of 9 months (range 6–30).

### Clinical characteristics

We collected the following clinical and laboratory data: baseline characteristics (age at disease diagnosis, age at Obinutuzumab start, sex, comorbidities, renal biopsy, pathological stage, previous immunosuppressants) and clinical data (number of doses with Obinutuzumab, 24-h urine protein, serum albumin, blood creatinine, eGFR, total cholesterol, anti-phospholipase A2 receptor antibody, CD19+ B-lymphocyte count, follow-up time). All data were systematically got via the hospital’s electronic medical record systems and telephone follow-up visit. PLA2R-Ab was detected via enzyme-linked immunosorbent assay (Euroimmun, threshold:0-20 R U/mL). This study was approved by the Ethics Committee of the Second Hospital of Hebei Medical University (2026-R240) and was conducted in accordance with the principles of the Declaration of Helsinki. Informed consent has been obtained from all participants.

### Treatment protocol

Obinutuzumab was administered as two separate 1 g intravenous infusions over two to four weeks, with a subsequent 1 g infusion at approximately six months in most patients. The treatment regimen was determined by the medical team based on the patient’s clinical condition and laboratory findings. Dissolve 1 g of Obinutuzumab in 500 mL of saline. The infusion was monitored at a rate of 25 mL/h within the initial 30 min, the rate being gradually increased every 60 min to the goal of 100 mL/h, depending on patient tolerance. To minimize infusion reactions, 1 h before the infusion, 80 mg of methylprednisolone was administered intravenously and 20 mg of diphenhydramine was injected intramuscularly.

We evaluated all patients before Obinutuzumab infusion to exclude patients who had contraindications. Routine blood parameters, electrolytes, cardiac enzymes, troponin, chest computed tomography images and ECG findings were collected prior to infusion.

### Definitions

The outcomes were clinical remission of proteinuria and immunological remission.

Complete remission (CR) is defined as proteinuria <0.3 g/24 h in the absence of kidney function deterioration along with normal serum albumin.

Partial remission (PR) is defined as proteinuria <3.5 g/24 h and a urine protein reduction of at least 50 % compared to baseline, with stable kidney function and albumin rising.

Immunological remission is defined as the level of antiphospholipase A2 receptor (PLA2R) antibody <2RU/mL in PLA2R-associated primary membranous nephropathy [15].

Recurrence is defined as 24 h urine protein greater than 3.5 g/day again after CR or PR.

B- cell reconstitution is defined as absolute CD19-positive cell count >5 cells/μL through peripheral blood flow cytometry.

Rituximab-associated recurrence was defined as the recurrence of nephrotic syndrome after complete or partial remission, despite the administration of a standard rituximab regimen consisting of two intravenous infusions of 1,000 mg given 14 days apart.

Rituximab-resistance was defined as non-responders (failed to induce immunological and clinical remission)or experiencing disease progression despite the standard treatment regimen of rituximab.

Side effects associated with Obinutuzumab include infusion-related reactions and those occurring during follow-up. Serious side effects are defined as those requiring hospitalization or resulting in significant organ dysfunction.

### Statistical analysis

Statistical software SPSS 23.0 and Prism software were used for analysis and chart. Parametric data are presented as mean ± standard deviation (SD), and nonparametric data are presented as median (range). Categorical data were presented as number (percentage). Longitudinal changes in proteinuria, serum albumin, serum creatinine, eGFR and PLA2R-ab were analyzed using a linear mixed-effects model to account for within-subject correlations. Time (month) was included as a fixed categorical effect. A random intercept for each subject was incorporated to model individual baseline variations. The model’s goodness-of-fit was assessed using maximum likelihood estimation. Statistical significance was set at p<0.05. Statistical analyses of the Chi-square test and the Chi-square test were performed to investigate the association between different patient groups (e.g., responders vs. non-responders to Obinutuzumab) and baseline characteristics or clinical parameters.

### Research ethics

This study was approved by the Ethics Committee of the Second Hospital of Hebei Medical University (2024-R399) and was conducted in accordance with the principles of the Declaration of Helsinki. The ethical approval was granted on June 17, 2024.

### Informed consent

Informed consent was obtained from all individuals included in this study.

## Results

### Baseline characteristics

Baseline characteristics of the study population are summarized in the Table 1 below. Of the 25 patients, 17 were men, constituting 68 % of the study population. The mean age at disease diagnosis was 43.3 ± 12.8 years and the mean age at the start of Obinutuzumab treatment was 46.5 ± 12.0 years. Prior to Obinutuzumab treatment, the comorbidities included: primary hypertension (n=13), diabetes mellitus (n=5), and concomitant coronary and cerebrovascular disease (n=4). Renal biopsy performed in all patients demonstrated pathological findings consistent with PMN at various stages, in some cases combined with other kidney disease. Patients with CKD stages 1–4 were48 %, 24 %, 20 % and 8 % respectively. Prior to Obinutuzumab therapy, the patients had received the following immunosuppressive regimens: A total of eight patients had previously received glucocorticoids plus cyclophosphamide, and 18 patients had received calcineurin inhibitors The majority of them were given two or more immunosuppressive agents. All 25 patients had been treated with rituximab. 20 patients were identified as rituximab-resistant, while five had recurrent disease after an initial response. The median intervals between Obinutuzumab and rituximab treatments were six months (range 4–22). Among the patients treated with Obinutuzumab, the majority (84 %, n=21) received three 1-g infusions. Elevated serum anti-PLA2R antibody levels were observed in 21 patients, with a median level of 133.85 RU/mL (range 10.88–820.41). Key baseline data included a 24-h urine protein level of 11.3 ± 5.6 g, serum albumin of 21.7 ± 5.5 g/L, serum creatinine of 81 (median; range 29–370) μ mol/L, and an eGFR of 81.1 ± 34.1 mL/min/1.73 m^2^. Other parameters were total cholesterol (6.46 ± 1.77 mmol/L) and CD19+ B cell count (111 ± 84/μ L), with a mean follow-up of 9 months (range, 6–30).

**Table 1:** Baseline characteristics of the study population.

Characteristics	Patients of primary membranous nephropathy
Age years, mean ± SD	
Age at disease diagnosis	43.3 ± 12.8
Age at obinutuzumab start	46.5 ± 12.0
Sex ratio (F/M)	8/17
Comorbidities	
Diabetes, n (%)	5 (20 %)
Hypertension, n (%)	13 (52 %)
Coronary and cerebrovascular diseases	4 (16 %)
Pathological findings Pathological stage of MN, n (%)	
I	15 (60 %)
II	6 (24 %)
III+IV	0 (0 %)
Atypical membranous nephropathy	4 (16 %)
Combination of other pathological types	
Combined hypertensive kidney damage	4 (16 %)
Combined diabetic kidney disease	1 (4 %)
Combined IgA nephropathy	1 (4 %)
CKD stage, n (%)	
I	12 (48 %)
II	6 (24 %)
III	5 (20 %)
IV	2 (8 %)
Previous IS treatments, n (%)	
Glucocorticosteroid + cyclophosphamide	8 (32 %)
Calcineurin inhibitor ± glucocorticosteroid	18 (72 %)
RTX	25 (100 %)
Two or more immunosuppressants	20 (80 %)
Obinutuzumab, number of doses, n (%)	
Three	21 (84 %)
Four	3 (12 %)
Five	1 (4 %)
Anti-PLA2R antibody	
Positive history, n (%)	21 (84 %)
Serum levels, RU/mL, Median (IQR)	133.85 (10.88–820.41)
24-h urinary protein excretion, g, mean ± SD	11.3 ± 5.6
Serum albumin, g/L, mean ± SD	21.7 ± 5.5
Serum creatinine, µmol/L, median (IQR)	81 (29–370)
eGFR (CKD-EPI), mL/min/1.73 m^2^, Mean ± SD	81.1 ± 34.1
CHOL, mmol/L, Mean ± SD	6.46 ± 1.77
CD19 positive B-cells, number/μl, mean ± SD	111 ± 84
Follow-up time, months, median (IQR)	9 (6–30)

Quantitative data were expressed as median (minimum, maximum) or mean ± standard deviation (SD). Categorical data were presented as frequencies. eGFR, estimated glomerular filtration rate; IS, immunosuppressant; PLA2R-Ab, antiphospholipase A2 receptor antibody; MN, membranous nephropathy; RTX, rituximab; CKD, chronic kidney disease.

### Clinical outcomes

Complete or partial remission of proteinuria was achieved in 20 of the 25 patients (80 %), with 4 achieving CR and 16 achieving PR during the follow-up period (Figure 1a). All four patients who achieved complete remission had previously attained partial remission. The remission rates increased over time. At the 1-month follow-up, 3 patients (12 %) achieved partial remission. This increased to 8 patients (32 %) at 3 months and further to 15 patients (60 %) at 6 months. At the end of follow-up, clinical remission of proteinuria was achieved in 20 patients (80 %). The time to achieve clinical remission was 4 months (range 1–14). Renal function remained stable in all patients. Among the remaining five patients who did not achieve remission, the clinical outcomes varied: two showed a >50 % reduction in urinary protein excretion from baseline; one presented with nephrotic-range proteinuria despite normalized serum albumin levels; and two demonstrated no therapeutic response (non-responders). We explored the relationship between response to Obinutuzumab and patients’ baseline characteristics and clinical indicators. Analysis of responders (n=20) and non-responders (n=5) indicated that higher baseline proteinuria and elevated serum creatinine were associated with a poorer response to Obinutuzumab (Supplementary Fig1a.b). The baseline antiphospholipase A2 receptor antibody (PLA2R-Ab) levels in non-responders and responders of Obinutuzumab were no statistically significant difference (p=0.455).

**Figure 1a:**
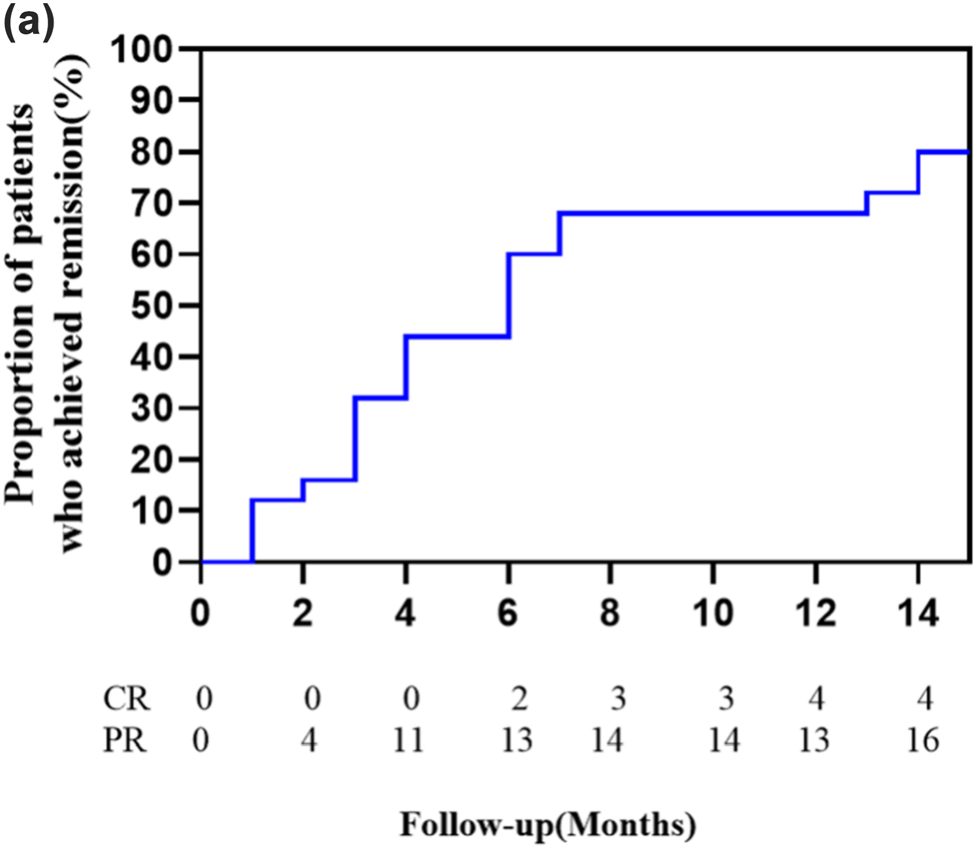
Proportion of patients achieving complete or partial remission of proteinuria. Using Prism software, we generated Kaplan–Meier survival curves to visualize the proportion of patients achieving remission of proteinuria. CR, complete remission; PR, partial remission. The number under each month represents the total number of patients who experienced remission.

Based on their response to rituximab, we categorized them into a rituximab-resistant group (n=20) and a rituximab-recurrent group (n=5). Remission rates differed between the groups: 15 patients (75 % of the resistant group) achieved proteinuria remission, in contrast to all five patients (100 %) in the rituximab-recurrent group (Figure 1b). The difference was not statistically significant in the two groups.

**Figure 1b:**
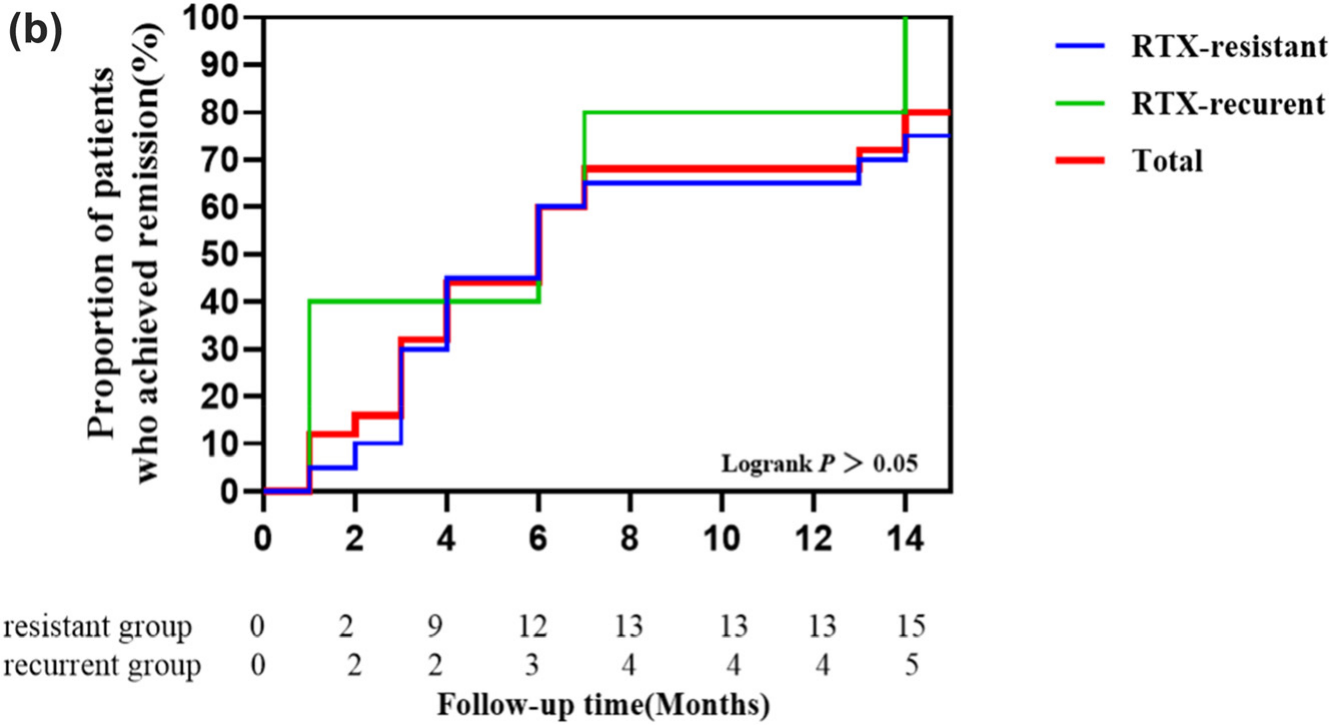
Proportion of patients achieving remission of proteinuria in RTX-resistant group and RTX-recurrent group. Using Prism software, we generated Kaplan–Meier survival curves to visualize proportion of patients achieving remission of proteinuria in RTX-resistant group and RTX-recurrent group. The number under each month represents the total number of patients who experienced remission in different group.

Changes in 24-h urine protein, serum albumin, serum creatinine and eGFR were analyzed using a linear mixed-effects model (Figure 2a, 2b, 2c, 2d).

**Figure 2a:**
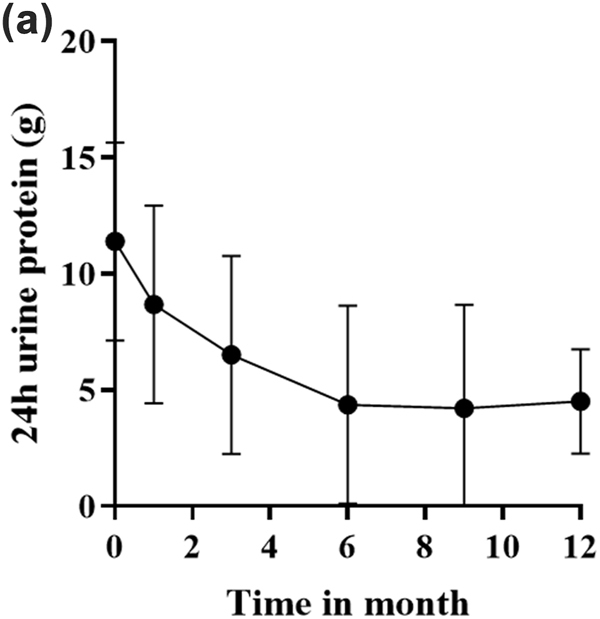
Longitudinal changes in proteinuria. Data are estimated marginal means from a linear mixed-effects model with 95 % confidence intervals. The overall effect of time was significant. The values in the figure are presented as the mean ± 95 % confidence interval.

**Figure 2b:**
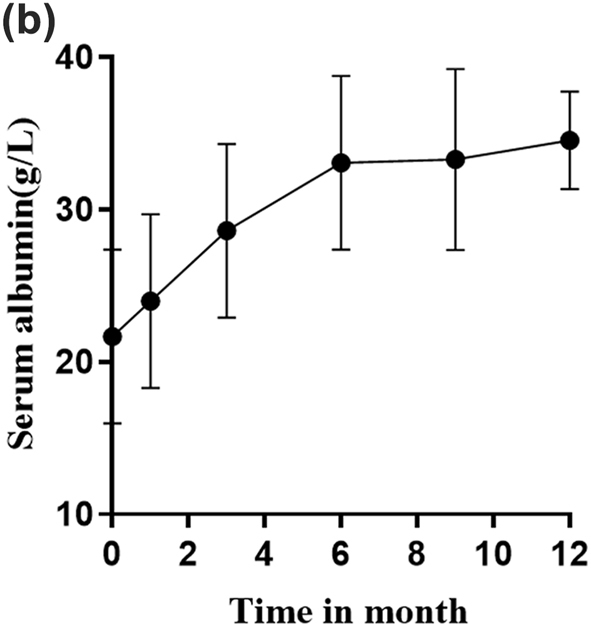
Longitudinal changes in serum albumin. Data are estimated marginal means from a linear mixed-effects model with 95 % confidence intervals. The overall effect of time was significant. The values in the figure are presented as the mean ± 95 % confidence interval.

**Figure 2c:**
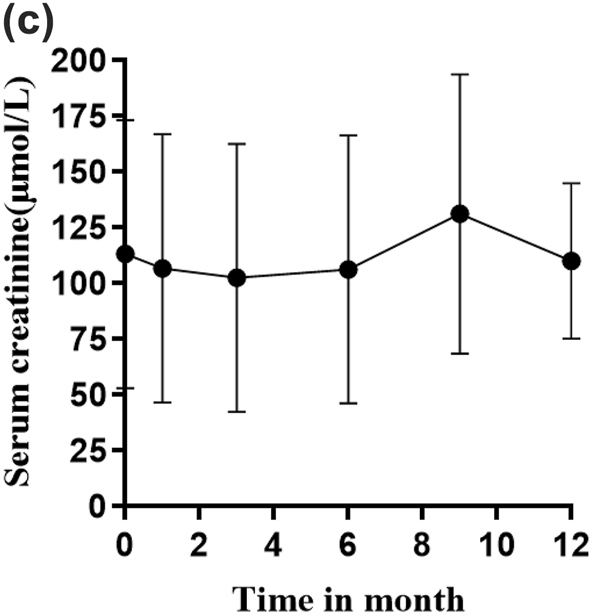
Longitudinal changes in serum creatinine. Data are estimated marginal means from a linear mixed-effects model with 95 % confidence intervals. The overall effect of time on serum creatinine levels was not significant. The values in the figure are presented as the mean ± 95 % confidence interval.

**Figure 2d:**
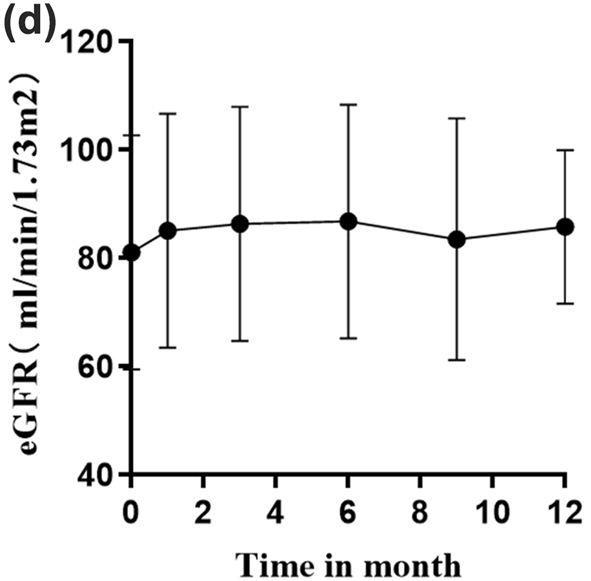
Longitudinal changes in eGFR. eGFR were measured at baseline and at 1, 3, 6, 9, and 12 months following obinutuzumab administration. The figure showed the median and range. The values in the figure are presented as the mean ± 95 % confidence interval. eGFR, estimated glomerular filtration rate.

The model revealed a statistically significant overall effect of time on proteinuria levels (p<0.001). Post-hoc comparisons, using the 12-month value as the reference, demonstrated that proteinuria was significantly elevated at baseline (p<0.001), month 1 (p<0.001), and showed a trend towards elevation at month 3 (p=0.052). In contrast, proteinuria levels at month 6 (p=0.89) and month 9 (p=0.80) were not statistically different from those at the final 12-month assessment (Figure 2a).

A highly significant overall effect of time on albumin levels was observed (p<0.001). When compared to the 12-month reference level, serum albumin was significantly lower at baseline (p<0.001), month 1 (p<0.001), and month 3 (p<0.001). No statistically significant differences from the 12-month level were detected at month 6 (p=0.24) or month 9 (p=0.36) (Figure 2b).

The analysis revealed no statistically significant overall effect of time on serum creatinine levels (p=0.264). Post-hoc comparisons against the 12-month reference value showed no significant differences at any individual time point: baseline (p=0.81), month 1 (p=0.80), month 3 (p=0.56), month 6 (p=0.77), or month 9 (p=0.13) (Figure 2c).

The analysis indicated no statistically significant overall change in eGFR levels over time (p=0.369). Post-hoc comparisons against the 12-month value revealed no significant differences at any assessed time point, including baseline (p=0.21),month 1 (p=0.85),month 3 (p=0.88), month 6 (p=0.79), or month 9 (p=0.58) (Figure 2d).

### Serum PLA2R antibody

In the 21 patients with PLA2R-associated primary membranous nephropathy, 13 patients (62 %) achieved immunologic remission concurrently during the follow-up time. Immunologic remission was attained by four at one month and by eight at three months of Obinutuzumab therapy. (Figure 3a). Figure 3b showed the specific measurement values in the 21 patients with PLA2R-Ab associated MN. Notably, among the eight patients classified as non-responders based on immunological criteria, six patients exhibited a significant reduction in antibody titers. The remaining two patients experienced an initial decline followed by a titer rebound during follow-up. Changes in PLA2R-Ab titers were analyzed using a linear mixed-effects model The model revealed a highly significant overall effect of time on antibody levels (p<0.001). Post-hoc comparisons, with the 6-month time point as the reference, demonstrated that antibody levels were substantially and significantly higher at baseline compared to month 6 (p<0.001). In contrast, antibody levels at month 1 (p=0.29) and month 3 (p=0.78) were not statistically different from those at month 6 (Figure 3c). No significant association was observed between the longitudinal trajectory of anti-PLA2R antibody levels and the change in proteinuria over time.

**Figure 3a:**
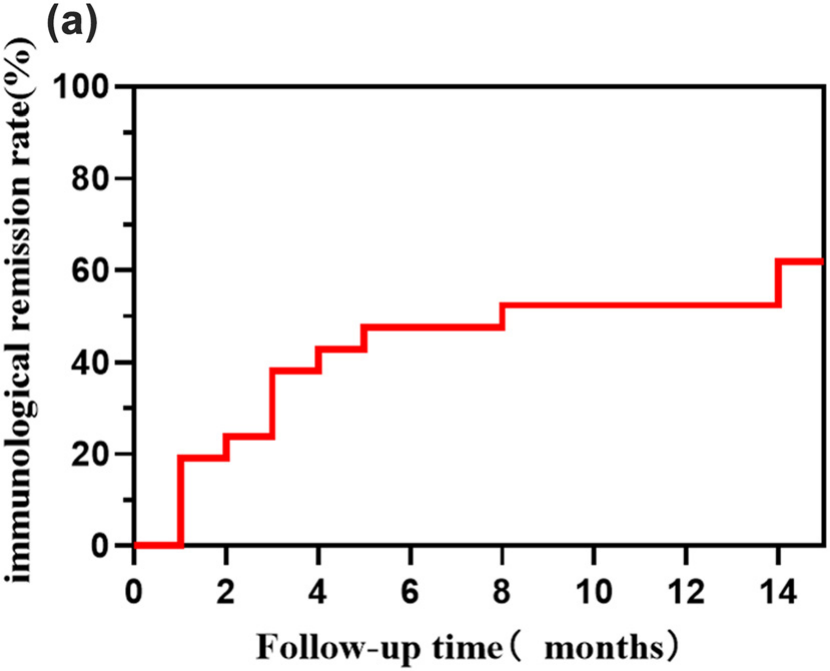
Immunological remission in PLA2R-Ab positive patients. Using Prism software, we generated Kaplan–Meier survival curves to visualize the proportion of patients achieving immunologic remission.

**Figure 3b:**
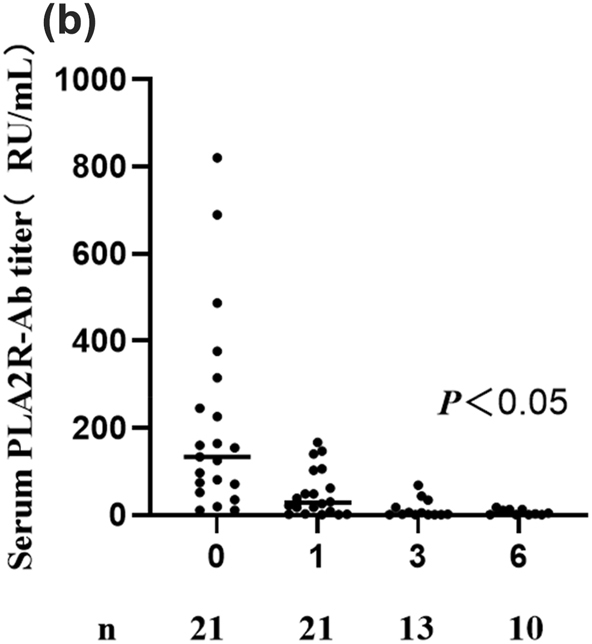
Serum PLA2R-Ab in PLA2R-Ab positive patients. Serum PLA2R-Ab levels were measured at baseline and at 1, 3 and 6 months following obinutuzumab administration.

**Figure 3c:**
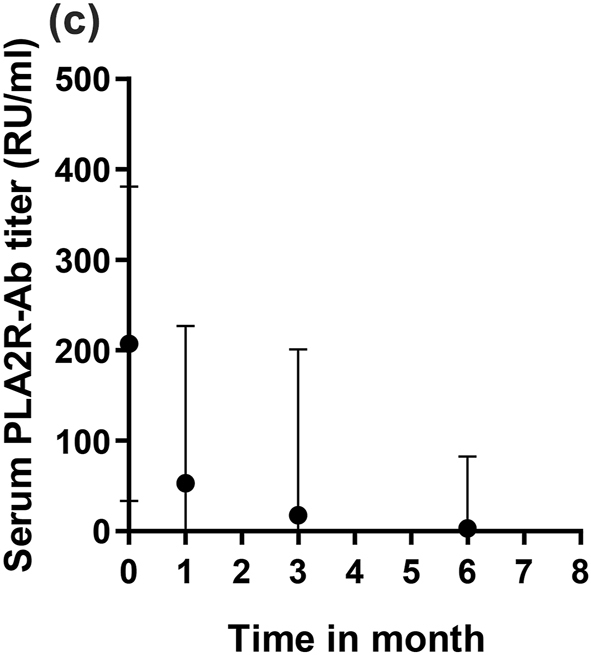
Longitudinal changes in PLA2R-Ab levels data are estimated marginal means from a linear mixed-effects model, with 95 % confidence intervals (CIs). Because PLA2R antibody titers are biologically non-negative, the lower limits of the CIs were truncated at zero. The overall effect of time was significant. Values are presented as mean ± 95 % CI.

### CD19+ B-cells

All patients underwent CD19+B-cell count testing before infusion of Obinutuzumab which was 111 ± 84/μL. Within two to four weeks following the initial infusion, we re-evaluated CD19+ B-cell count. The results showed that CD19+B-cells were fully depleted. Reconstitution of CD19+B-cell occurred more than 6 months (range 5–9) after Obinutuzumab infusion.

### Relapse and correlation analysis of data

During the entire follow-up period, relapse occurred in two patients, both of whom had been off medication for nine months. One patient was in the rituximab-recurrent group and the other patient was in the rituximab-resistant group. With another additional dose of Obinutuzumab, both of them achieved partial remission. No significant associations were observed between disease recurrence and the examined variables, including gender, age at onset, pre-treatment antibody levels, pre-treatment CD19+B-cell count and renal pathology. A longer interval between the initial treatment and the subsequent administration of Obinutuzumab is associated with an increased risk of disease recurrence.

### Severe adverse effects

Obinutuzumab was well tolerated in most patients. All adverse events were mild to moderate and reversible. No severe adverse events were recorded during or after Obinutuzumab infusion. Two patients experienced chills during infusion, which was resolved with a second dose of 40 mg of methylprednisolone, slowing the infusion rate. Three patients experienced transient skin rash on the body and one patient had thoracic pain which improved after symptomatic treatment. No infection-related diseases appeared.

## Discussion

With the recognition of membranous nephropathy as an autoimmune disease, targeting B-cells has emerged as an effective therapeutic agent for PMN [19]. Obinutuzumab is considered effective and safe for the treatment of membranous nephropathy [13], [14], [15], [16], [17], [18, [20], [21], [22], [23]. Our research aligned with this conclusion. We retrospectively analyzed the efficacy and safety of Obinutuzumab in treating 25 patients with rituximab-resistant and rituximab-recurrent membranous nephropathy with a median follow-up of 9 months (range 6.0–30.0). After six months of treatment with Obinutuzumab, 15 patients (60 %) achieved remission. At the end of the follow-up period, 20 patients (80 %) had achieved remission, comprising 16 with partial remission (PR) (64 %) and 4 (16 %) with complete remission (CR). Among the remaining five patients who did not achieve remission, the clinical outcomes varied: two showed a >50 % reduction in urinary protein excretion from baseline; one presented with nephrotic-range proteinuria despite normalized serum albumin levels; and two demonstrated no therapeutic response (non-responders). In Supreet Sethi’s study of Obinutuzumab [21], complete remission was achieved in 4 (40 %) and partial remission was achieved in 5 (50 %) of patients after 6 months administer of Obinutuzumab. Among these five patients, sustained remission was maintained throughout the 24-month follow-up period. A report of three cases of PLA2R-associated membranous nephropathy refractory to rituximab demonstrated that two patients achieved immunological remission within six months of Obinutuzumab treatment, accompanied by improved proteinuria and a reduction in anti-PLA2R antibody titers [15]. In our study, the six-month remission rate was lower than those reported in previous studies, which may be attributed to differences in patient selection. The majority of our patients had previously failed treatment with two or three immunosuppressive agents, highlighting the refractory nature of their condition. Additionally, some patients in the study exhibited persistent proteinuria despite normal plasma albumin levels, which were classified as having partial remission. This may be one of the reasons for the low rate of complete remission. The time to remission was four months (range1–14) in our study. In a research of PLA2R-associated membranous nephropathy, the time to complete remission varied among patients, with a duration of one to nine months [16].

Multiple studies have demonstrated a strong association between anti-PLA2R antibody titers and proteinuria, with the prevailing view that immunological remission precedes clinical remission of proteinuria [24], [25], [26]. Among the 21 patients with PLA2R-associated primary membranous nephropathy, 13 achieved concurrent immunologic remission. Our results showed a highly significant overall effect of time on antibody levels. But longitudinal trajectory of PLA2R-Ab levels had no relationship with the change in proteinuria over time.

For patients with MN who have previously failed to respond to rituximab therapy, treatment with Obinutuzumab may represent a potential therapeutic alternative. In our cohort, proteinuria remission was achieved in 75 % (15/20) of rituximab-resistant patients, among whom 50 % (10/20) also attained immunologic remission. Similarly, in a trial of 18 refractory membranous nephropathy patients treated with Obinutuzumab [17], 17 patients (94.4 %) achieved either PR or CR, showing efficacy even in 11 patients (61.1 %) with unsatisfactory responses to previous rituximab treatments.

The higher efficacy of Obinutuzumab in the treatment of refractory membranous nephropathy has been linked to its potent and durable clearance of B cells. Obinutuzumab is a humanized and glycoengineered type II anti-CD20 monoclonal antibody which consists of a non-human hypervariable region, but a human framework and a human constant region [27]. On the one hand, Obinutuzumab was designed glycol engineered. This results in an increased affinity of the drug for FcγRIIIa on B cells, which leads to an elevated ability to bind and recruit effector cells, resulting in higher antibody-dependent cell cytotoxicity and less dependence on complement-mediated activity compared to rituximab [11]. On the other hand, Obinutuzumab is designed to bind an overlapping epitope of CD20 in a different orientation. This modification prevents internalization of the CD20/antibody complex. Obinutuzumab also induces direct lysosomal-mediated cell death and is less dependent on high levels of B-cell activating factor, contributing to greater depletion of memory B cells classically more resistant to rituximab [9], 28], 29]. Obinutuzumab also eliminates B cells from the lymph nodes and had lower immunogenicity risk [30]. Among the 5 patients in the rituximab-recurrent group, the treatment with Obinutuzumab maintained a longer duration of complete B-cell depletion compared to the rituximab. Reconstitution of CD19+B-cells occurred more than 6 [5], [6], [7], [8], [9] months after infusion. This observation was consistent with findings from previous studies [11], 21]. But the lack of regular monitoring of peripheral lymphocyte counts and the small number of participants introduces bias into the assessment of the duration of B cell depletion (BCD) maintenance. No conclusions could be drawn regarding the potential relationship between B-cell depletion or reconstitution and the incidence of proteinuria in our research. Ofatumumab is regarded as fully human anti-CD20 monoclonal antibody which contains fully human immunoglobulin. In contrast, it has a response rate of only 3/10 in patients with membranous nephropathy who are resistant to rituximab [31].

Relapse occurred in two patients in our study. No significant associations were observed between disease recurrence and the examined variables, including gender, age at onset, pre-treatment antibody levels, pre-treatment CD19+B-cell count and renal pathology. A longer interval between the initial treatment and the subsequent administration of Obinutuzumab is associated with an increased risk of disease recurrence. The limited sample size and relatively short follow-up period in our study hinder the ability to establish a causal relationship between infusion intervals and recurrence risk. Therefore, larger, long-term studies are required to confirm this association. Moreover, we conducted a correlation analysis of response to Obinutuzumab. Higher baseline proteinuria and serum creatinine levels were associated with a poorer response to Obinutuzumab. These findings appear to suggest that among patients who have already met the criteria for medication, earlier initiation of Obinutuzumab treatment – as opposed to delaying until the progression of proteinuria or a further increase in serum creatinine levels – is associated with improved clinical outcomes. However, due to limitations such as the small number of studies, this conclusion still requires further exploration.

Among the seven patients with severe renal impairment (CKD stage 3–4), four achieved partial remission. One patient had already attained a reduction in urinary protein exceeding 50 % from baseline accompanied by a significant increase in serum albumin. The remaining two patients were non-responsive to Obinutuzumab and exhibited deterioration in renal function. There were no explanations for the deterioration in renal function and another renal biopsy was proposed. Both patients declined our recommendation. In most patients with severe renal impairment, renal function was improved or stabilized, demonstrating the safety and efficacy of Obinutuzumab treatment in this population, which consistent with that proposed by Naik S et al. [23].

Our data revealed that urinary protein levels had a rebound increase at the 12-month follow-up point after Obinutuzumab administration. We have conducted a thorough investigation into the potential underlying causes of this phenomenon. The number of patients at the follow-up decreased. The values appeared to be skewed. Some patients achieved proteinuria remission who did not reach the later monitoring points. The absence of this group with lower protein levels may influence the results. There were no significant differences in renal function over time.

A subset of patients demonstrated the persistence of proteinuria, with some manifesting nephrotic-range levels, despite achieving normalized serum albumin. It was also found in a case report published by Rebecca Hudson on the treatment of phospholipase A2-associated membranous nephropathy with obinutuzumab [14]. We hypothesize that this may be related to the fact that the patients develop focal segmental glomerulosclerosis. The residual proteinuria in MN has been shown in animal models to be secondary to the remodeling of the glomerular basement membrane, resulting in an altered architecture of the podocytes [32].

In summary, our findings support the established efficacy and safety of Obinutuzumab in primary membranous nephropathy, while also providing novel insights. Firstly, higher baseline proteinuria (p=0.001) and serum creatinine levels (p=0.03) were associated with a poorer response. Secondly, our cohort included patients across CKD stages 1 to 4, permitting an assessment of Obinutuzumab’s efficacy and safety profile in a broadly representative population. However, several limitations of this study should be acknowledged. Its retrospective, single-center design and the absence of a control or comparison group constrain the generalizability of the findings. Our relatively short follow-up would underestimate the clinical and immunologic remission of membranous nephropathy with Obinutuzumab treatment. True B-cell reconstitution might take longer. All MN patients included in our study were identified based on initial biopsy. Whether these patients might progress to FSGS remains unknown.

## Conclusions

Obinutuzumab may represent a promising treatment strategy for rituximab-resistant and recurrent membranous nephropathy, including in cases with severe renal impairment. A poorer response to obinutuzumab may be associated with higher baseline proteinuria and serum creatinine levels.

## Supplementary Material

Supplementary Material
