# Supplementary material for: Efficacy and safety of obinutuzumab as rescue therapy for rituximab-resistant and rituximab-recurrent primary membranous nephropathy: a retrospective cohort study
Source: Open Med (Wars). 2026 Jun 15;21(1):20261440. doi: 10.1515/med-2026-1440 (PMC13268011; doi:10.1515/med-2026-1440)
Supplement: Supplementary file 1 — Supplementary Material [file j_med-2026-1440_suppl_001.docx]

**Supplementary Fig.1a.1b**

Based on patient response to Obinutuzumab, they can be categorized into responders and non-responders.

We compared differences in urinary protein and serum creatinine levels between the responsive group and the non-responsive group.
